# Demographics, epidemiology and the impact of vaccination campaigns in a measles-free world – Can elimination be maintained?

**DOI:** 10.1016/j.vaccine.2017.02.008

**Published:** 2017-03-13

**Authors:** J.M. Prada, C.J.E. Metcalf, S. Takahashi, J. Lessler, A.J. Tatem, M. Ferrari

**Affiliations:** a Department of Ecology and Evolutionary Biology, Princeton University, USA; b Office of Population Research, WWS, Princeton University, USA; c Fogarty International Center, National Institutes of Health, USA; d Department of Epidemiology, Johns Hopkins Bloomberg School of Public Health, Baltimore, USA; e WorldPop, Department of Geography and Environment, University of Southampton, UK; f Flowminder Foundation, Stockholm, Sweden; g Center for Infectious Disease Dynamics, Pennsylvania State University, USA

**Keywords:** Measles, Epidemiology, SIR, Mathematical models, Vaccines

## Abstract

**Introduction:**

All six WHO regions currently have goals for measles elimination by 2020. Measles vaccination is delivered via routine immunization programmes, which in most sub-Saharan African countries reach children around 9 months of age, and supplementary immunization activities (SIAs), which target a wider age range at multi-annual intervals. In the absence of endemic measles circulation, the proportion of individuals susceptible to measles will gradually increase through accumulation of new unvaccinated individuals in each birth cohort, increasing the risk of an epidemic. The impact of SIAs and the financial investment they require, depend on coverage and target age range.

**Materials and methods:**

We evaluated the impact of target population age range for periodic SIAs, evaluating outcomes for two different levels of coverage, using a demographic and epidemiological model adapted to reflect populations in 4 sub-Saharan African countries.

**Results:**

We found that a single SIA can maintain elimination over short time-scales, even with low routine coverage. However, maintaining elimination for more than a few years is difficult, even with large (high coverage/wide age range) recurrent SIAs, due to the build-up of susceptible individuals. Across the demographic and vaccination contexts investigated, expanding SIAs to target individuals over 10 years did not significantly reduce outbreak risk.

**Conclusions:**

Elimination was not maintained in the contexts we evaluated without a second opportunity for vaccination. In the absence of an expanded routine program, SIAs provide a powerful option for providing this second dose. We show that a single high coverage SIA can deliver most key benefits in terms of maintaining elimination, with follow-up campaigns potentially requiring smaller investments. This makes post-campaign evaluation of coverage increasingly relevant to correctly assess future outbreak risk.

## 1 Introduction

The high case fatality rates associated with measles in children, and the existence of an inexpensive, safe vaccine, have led measles control to be called a ‘best buy’ in public health [1]. Measles vaccine coverage has increased globally over the last decade, and vaccination is estimated to have averted 13.8 million deaths between 2000 and 2012 [2]. This success has led to proposals for a global effort to achieve measles eradication [3], [4], [5]. Reduction of global prevalence to zero requires that measles endemic areas achieve elimination, and that elimination be maintained in areas where measles is absent. Formally, this requires reducing the local effective reproduction number, *R_E_*, or number of new infections per infectious individual, below one and maintaining it at that level. Where *R_E_* is below one and measles has been eliminated, by definition, re-introduction of measles will not result in re-establishment of endemic transmission; however, small to mid-sized outbreaks may occur if measles is introduced.

The herd immunity threshold for the elimination of an immunizing pathogen is a central concept in infectious disease epidemiology and public health. The simplest result, ignoring maternal immunity, states that *R_E_* can be maintained below one and a pathogen can be eliminated if a fraction greater than, *p_c_* = 1–1/*R*_0_, where *R*_0_ is the basic reproduction number, is immunized by vaccination at birth. For pathogens with large *R*_0_, such as measles, this threshold can be difficult to achieve with only a single dose of childhood vaccine; even where routine first dose coverage is high, primary vaccine failure, ∼14% at 9 months, due to interactions with maternal immunity can limit immunization below the *p_c_* threshold [6]. Areas that have successfully eliminated measles, such as the countries in the Americas, have maintained their success through the introduction of a routine second vaccine dose. In many countries where measles remains endemic, population immunity is maintained through a combination of a single routine childhood dose and periodic, age-targeted supplemental immunization activities (SIAs). This approach necessarily means that population immunity will fluctuate over time. The proportion immune in the population will decrease in the interval between SIAs [7] as non-immunized susceptibles accumulate [8]. Thus epidemic risk, which scales as a non-linear function of the size and age distribution of the non-immune population [9], will increase. If infection is re-introduced when *R_E_* is transiently greater than one, both the likelihood that the initial infection will lead to an epidemic and the size of the resulting epidemic, increase as a non-linear function of *R_E_*.

Here we consider the ability of combined one-dose routine plus SIA vaccination strategies to maintain elimination once achieved. In the absence of circulating transmission, routine vaccination controls the rate at which susceptible individuals are recruited into the population and SIAs cause a periodic, pulsed reduction of susceptibles within certain age cohorts. Thus, optimization of maintenance strategies that involve SIAs should consider both the average population immunity attained, and the increased outbreak risk during transient periods of low population immunity, since the penalty of even a brief interval with *R_E_* > 1 may be a large outbreak. We evaluate vaccination strategies relative to an operational utility function, which we call integrated epidemic risk (IER - see methods below), that accounts for this non-linear change in outbreak risk as a function of the magnitude of *R_E_*.

Though many countries are far from local elimination of measles, some have made dramatic improvements in measles reduction and may be candidates for elimination in the near term [2]. To achieve the larger goal of regional elimination or global eradication, successful countries will need to maintain elimination while the other countries catch up. Thus, evaluating the potential for the current one-dose routine plus SIA vaccination strategies to maintain elimination is critical to planning for future measles vaccine investments. We explore the ability of a range of SIA designs to maintain elimination with scenarios designed to broadly reflect four sub-Saharan African countries, chosen to encompass a range of demographic and vaccination coverage scenarios. We illustrate that while broader age target SIAs necessarily reduce *R_E_* and the risk of outbreaks, the marginal benefits of these campaigns depend on the country context.

## 2 Materials and methods

We created an age structured epidemiological MSIRV (Maternally immune – Susceptible – Infected – Recovered – Vaccinated) compartmental model, following methods developed by Metcalf et al. [10]. We simulated the progression of the effective reproduction number, *R_E_*, and IER of measles in four different populations under the assumption of no circulating transmission. This requires five steps, detailed below: initializing the starting susceptible population size and distribution, forward simulation of the susceptible population through time, evaluation of *R_E_* at each time point, estimation of the integrated epidemic risk, and marginal benefit comparison.

We initialized populations to broadly reflect the demography of four countries in sub-Saharan Africa chosen to capture a diversity of demographic and vaccination contexts: (i) Ethiopia, with a large urban population and a relatively low historical routine vaccination rate; (ii) Nigeria, the most populated country in Africa and with a moderate historical vaccination rate; (iii) Equatorial Guinea, with a small population and a low vaccination level; and (iv) Swaziland, with a small population but a high vaccination level.

### 2.1 Initial populations

Initial population age distributions were based on estimates for each country from the WorldPop project [Ref. http://www.worldpop.org.uk/, Tatem et al. 2013] stratified into 225 age groups (monthly strata up to 15 years of age; yearly thereafter). In each age bin, the population is further divided into relevant epidemiological compartments (i.e., susceptible, recovered, vaccinated, etc). We estimated the initial population in each age group that was immunized by vaccination using UNICEF/WHO administrative coverage estimates since 1980 for routine and SIAs, combined with the efficacy of the vaccine (Supplementary Material 1). All individuals are assumed to be born with maternal immunity and its decay with age was modeled as a monthly exponential decline with a rate = 0.45, which translates into less than 5% maternally immune by 7 months [7]. The initial number of individuals with immunity from natural infection (“recovered” individuals) was estimated by assuming a constant hazard of infection with age; thus the probability of remaining susceptible declines as an exponential function of age. We assumed an exponential rate of 0.02, which is equivalent to assuming a mean age of infection of 4 years, but explored a range of values for this parameter (Supplementary Material 1). After allocating the corresponding proportion of the population to the 3 compartments above (V, M and R), the remainder were added to the susceptible compartment.

### 2.2 Forward simulation

We simulated the trajectory of the population forward for 15 years, from 2015 to 2030. Individuals age into the next age-class in each time step at a rate equal to one over the size of the age group, measured in bi-weekly time-steps (i.e. 1/2 for the monthly age bins, 1/24 for the yearly age bins). Birth rates were taken as the 2015 estimate from World Bank (“http://data.worldbank.org/”) and assumed constant throughout the simulation (alternative parametrisation in Supplementary Material 1). All infants are assumed to be born with maternal immunity, described above. Age-specific mortality was estimated by spline interpolating the data from the UN Department of Economic and Social Affairs model life tables (http://esa.un.org/unpd/wpp/Download/Standard/Mortality/), which is given in 5-year age bins, to our age groups.

Immunity through vaccination comes from two sources, routine coverage and SIAs. Routine vaccination coverage levels reflected those achieved in 2014 (the most recently available year in the WHO/Unicef database) and were assumed to remain constant over the simulation time-frame. For SIAs, we explored scenarios reflecting campaigns targeting 3 different age classes (9m to 5y, 9m to 10y, 9m to 15y) occurring every 4 years over a time period of 15 years. The frequency of campaigns reflected WHO recommendations from the global measles and rubella strategic plan [17]. Though the intent of campaigns is to vaccinate a large proportion of susceptibles in the target age class, the proportion of susceptibles immunized in these campaigns may differ from the vaccination coverage because of logistical constraints in implementation, under-estimation of the target population size, or heterogeneity in access to vaccination services [11]. Thus, we considered two levels of immunization achieved by these campaigns: 70% and 90%. In practice, many countries conduct so-called “catch-up” SIAs, which target a wide age range, followed by “follow-up” campaigns, which target a narrower range [12], and we also investigated the impact of this “catch-up, follow-up” strategy.

### 2.3 R_E_ and IER

Based on the projected size and age distribution of the susceptible population over time, we calculated *R_E_* at each simulation time step using the dominant eigenvalue of the next generation matrix [13]. Because contact patterns over age are unknown for the focal contexts, we assumed age-specific mixing based on the POLYMOD diary studies from different European countries [14]. The qualitative patterns indicated by POLYMOD are broadly consistent across Europe, and are echoed in a number of other settings [15], [16], and therefore likely to provide a reasonable approximation of local patterns; analysis with a flat contact matrix shown in Supplementary Material 1.

The effective reproduction number, *R_E_*, is a standard characterization of epidemic potential. However, expected outbreak size scales with *R_E_* in a non-linear fashion, so it does not fully capture the increased outbreak risk associated with *R_E_* values slightly above one. We define the Integrated Epidemic Risk, IER, as the expected outbreak size, divided by the total population, and averaged over the simulation time frame,

(1) $$\mathrm{IER}=\frac{1}{T}\overset{T}{\underset{\neq 0}{\sum }}\frac{C_{t}}{N_{t}}$$

where *C_t_* is the expected outbreak size after a single introduction at time *t* and allowing for the potential outbreak to develop over a maximum time of one year, *N_t_* is the population at the time of the introduction. We assume that the initial infectious individual is in the most transmitting age class at that time step (normally an individual younger than 15 years of age), as this represents the worst-case scenario.

### 2.4 Marginal benefit

Higher campaign coverage and wider age targets will both result in a greater reduction in susceptibles and lower IER. However, the marginal benefits of increasingly large campaigns should decrease once a campaign is of sufficient coverage to reduce *R_E_* to below one and because older individuals are more likely to be previously immunized. Thus we present the impact of larger campaigns in terms of the marginal benefit; i.e. reduction of IER per dose required to implement the campaigns, where the number of doses is used as a simple proxy for campaign cost. SIAs do not generally attempt to discriminate between immune and susceptible individuals within the target age range. As a result, previously vaccinated individuals (via routine vaccination or in a previous campaign) may be re-vaccinated. The minimum number of doses required for an SIA is thus defined by a combination of the number of individuals in the target age range, and the vaccination coverage the SIA achieves. The true cost of campaigns will depend on operational costs, as well as cost of vaccines and supplies; Vijayaraghavan et al. [17] estimated that vaccine cost and medical supplies account for around 65% of the total cost, while the rest are operational costs. A formal assessment of the operational costs of campaign deployment in each country is beyond the scope of this work, though such an analysis could be incorporated in the framework presented here.

## 3 Results

**Fig. 1:**
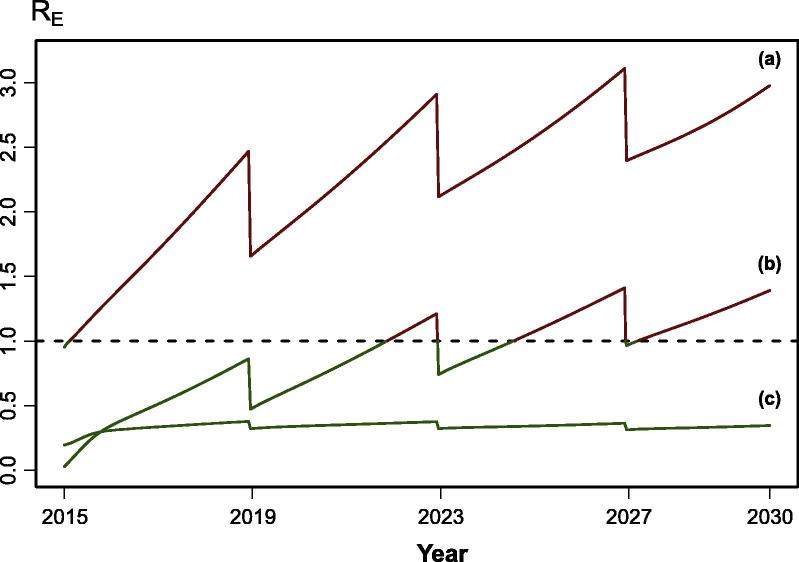
Possible R_E_ trajectories in each population; The drops in R_E_ reflect the implementation of a supplementary immunization activity (SIA). (a) shows a population dominated by the R_E_ > 1 regime, starting below 1 (elimination setting) but quickly increasing over one, without dropping even after the SIA campaign. (b) shows a population with increasing R_E_ that is reduced below 1 by the SIA campaigns, but large outbreaks can happen if an introduction occurs when the R_E_ is above 1. (c) is the ideal trajectory in an elimination setting, with the population's R_E_ always below 1, which prevents large outbreaks from happening.

The projected trajectory of *R_E_* exhibits one of 3 patterns: persistently *R_E_* *<* 1, persistently *R_E_* *>* 1, or *R_E_* fluctuating below and above 1 (Fig. 1). In all populations except the Swaziland-like, the default SIA campaign (70% coverage, up to 5 years) is not sufficient to prevent the *R_E_* trajectory from increasing above one over the 15 years evaluated (shading in Table 1). With increased campaign coverage and upper age range, populations spend more time in the *R_E_* *<* 1 regime. In the Equatorial Guinea-like population however, the *R_E_* value is above one in more than half of the time-steps, even in the scenario reflecting an SIA campaign that achieves high coverage (90%) and an upper age range of 15 years.

**Fig. 2:**
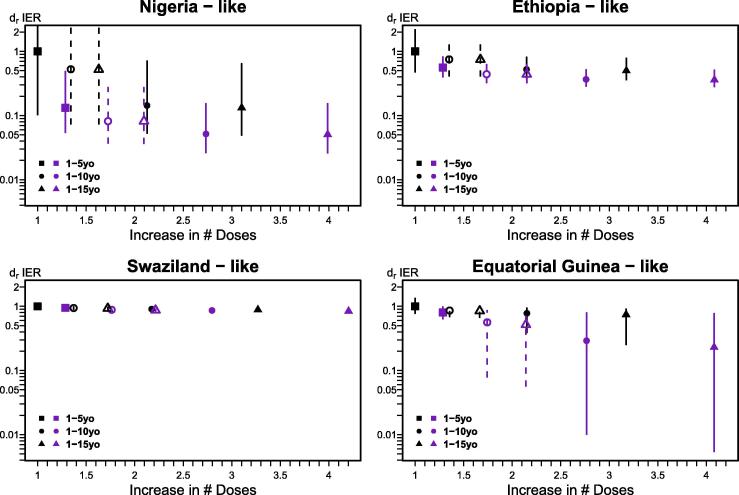
Relative difference in integrated epidemic risk (d_r_ IER) as the supplementary immunization activities (SIA) increase in coverage and target population in the four country-like urban populations modeled. The reference used is the default SIA campaign (70% coverage, target population up to 5 years). Vertical lines show the interquartile range. The y axis was logged and truncated in the Nigeria-like plot for easier interpretation of the results, in the default SIA scenario, outbreaks up to 14 times can happen in this population. Black lines indicate 70% coverage SIAs while purple lines show the results for 90% coverage. Full symbols and solid lines represent the SIAs with three equal campaigns, while the empty symbols and dashed lines represent the “catch-up”/“follow-up” setting.

Larger vaccination campaigns, either with wider target age ranges or higher immunization coverage, necessarily result in decreased *R_E_*, and therefore IER. However, because older individuals are more likely to be previously immunized and the relationship between epidemic size and *R_E_* is non-linear, the marginal benefit of increasing campaign impact (either coverage or age range) declines as their size increases (Fig. 2). The choice of campaign strategy impacts both the mean and inter-annual variability in risk (IER). In a Swaziland-like setting, for example, SIAs result in a small marginal increase in population immunity, but in a Nigeria-like setting, risk changes dramatically from lows following an SIA to highs prior to the subsequent SIA (vertical bars in Fig. 2).

Moreover, reducing R_E_ and IER comes at the cost of much larger investment as measured in doses required. Notably, a 90% coverage campaign targeting children <15 years requires >4 times as many doses as a campaign covering 70% of children <5 (Fig. 2) while increasing coverage requires fewer additional doses than increasing age range in all populations studied (Fig. 2). The reduction in IER is consistent across settings for high impact (90% immunization coverage) campaigns targeting children below 5 years, and low impact (70% immunization coverage) campaigns targeting children below 10 or 15 years (Fig. 2). Importantly, a campaign that achieves 90% coverage, but with an upper age range of only 10 years of age yields a very similar reduction in IER compared to the 15 year design in all four populations, while requiring considerably fewer doses (e.g., in the Nigeria-like population, 58.8 million doses across the 15 years – number of individuals vaccinated more than once in each scenario is shown in supplementary material 2).

Results reflecting a large “catch-up” SIA followed by two “follow-up” campaigns (targeting children below 5yrs of age) are summarized in Fig. 2 (dashed lines), showing similar trends to projections obtained with constant age (solid lines): IER decreases as the campaigns increase in coverage and age range (Table 2). In general, if SIA coverage is high (90%), the reduction in IER is similar in both settings and thus there is little marginal benefit of continued wide age campaigns.

## 4 Discussion

Even with very high vaccine coverage, routine vaccination at 9 months alone cannot maintain population immunity above the classic herd immunity threshold, as vaccine efficacy at this age is generally low. A combination of routine and pulsed, supplemental vaccination can be used to maintain high average levels of population immunity, but this strategy will necessarily result in *R_E_* increases in the periods between campaigns. Here we present an approach to evaluating the ability of these strategies to maintain high population immunity in areas where elimination has been achieved and minimize outbreak risk that integrates over these transient dynamics. We find that, for the strategies investigated here, SIAs often result in transient periods of *R_E_* below one, but can permit significant periods of *R_E_* *>* 1 in between campaigns depending on birth and routine vaccination rates. Thus, strategies for the maintenance of elimination in successful countries that rely on SIAs must account for local demographic conditions.

Wider campaigns necessarily reduce *R_E_* and IER, but the marginal benefit of expanding campaign age targets depends on the demographic context. In general however, in the scenarios studied here, SIAs targeting individuals up to 15 years do not significantly outperform (i.e. reduce IER significantly more) than SIAs targeting individuals up to 10 years, unless routine coverage is very low, as in our simulations in the Eq. Guinea-like population. If we account for dose deployment, high efficacy in maintaining elimination and avoidance of large outbreaks can be achieved with campaigns with high coverage (90%) and an upper age of 10 years, while maintaining a narrower age target (e.g., 5 years) results in campaigns that do not reduce susceptible numbers sufficiently to prevent large outbreaks (Fig. 2). Interestingly, these conclusions are broadly consistent across the range of contexts we evaluated.

Large campaigns may benefit from coordination of operational costs [17], while the marginal costs of reaching low-access populations may increase at high coverage [18] and higher coverage may be harder to achieve than a broader age range [19]; thus, full analysis of how campaign costs scale with immunization coverage achieved is complex, and likely to be context specific. We provide the most basic comparison of costs; minimum number of doses required. Importantly, by this metric, while the costs of the SIAs increases linearly with larger campaigns, the benefits in terms of reduction of IER saturate. Extension to consider further nuances is an important direction for future work.

Reducing the target age range of SIAs after the first one (i.e. catch-up / follow-up design), has a large benefit in reduction of doses needed overall, however, the outbreak risk can increase significantly, particularly if campaign coverage is low. The apparently counter intuitive results obtained in Equatorial Guinea-like populations, where the IER increases more at high coverage, emerge because low routine vaccination coverage means that low coverage SIAs all have little (and thus similar) effect in reducing risk, while the high coverage campaigns only have a significant effect when the target age is consistently wide (up to 10 or 15 years, Fig. 2 – bottom right). This raises a more general issue: across the settings investigated, much of the benefit can be achieved with a first campaign as long as the coverage achieved is high, so follow-up campaigns would potentially require smaller investments. Consequently, post-campaign evaluation becomes a priority, as it can inform the scope of future campaigns.

Though our characterization of outbreak risk following elimination is simplistic, it provides a tractable way to directly compare proposed campaign strategies across countries where the specific dynamics of local transmission might not be known. Our analysis assumes a single well-mixed population; which, although a conservative assumption, will lead to overestimation of outbreak size. Conversely, elimination is likely to be heterogeneous, and undetected cases may spark outbreaks in settings where elimination has been achieved [20]. Moreover, countries like Swaziland can have large in and out population movements making them less isolated closed populations. Deadlines for measles elimination goals in 5 WHO regions (all except SEAR) are approaching. Although major progress has been achieved in many settings [21], our framing emphasizes that achieving elimination is only the first step, and maintaining elimination may require substantial investments.

A second dose, provided via SIAs (as delineated here) or routine vaccination [6] emerges as key to maintaining elimination. The risk of future outbreaks, and measles re-invasion depends how the interaction between routine coverage and campaign age range and coverage shapes opportunities for vaccination. By characterizing this interaction, our analysis revealed two broad policy-relevant results: (i) campaign target ages extending beyond 10 years had little further marginal impact on epidemic risk, and (ii) increasing SIA coverage achieved tends to be more efficient than increasing age range, in situations where allocation decisions between the two have to be made.

## Conflict of interests

The authors declare no conflict of interests.

## Figures and Tables

**Table 1:** Integrated epidemic risk calculated as cases per million, *k* is used to symbolize 1000 cases per million, 95% credible interval in parenthesis. Shading of the cell represents the dynamic regime, white for *R_E_* always below one, dark gray for *R_E_* always above one and light gray for a mixed regime.

**Table 2:** Comparison between the 3 equal supplementary immunization activities settings and the “catch-up” - “follow-up” settings. Table shows the expected increase in percentage in integrated epidemic risk due to less vaccines being deployed in the latter setting. Campaign scenarios up to 5 years of age are the same in both settings, and thus are not shown.

Country	Coverage (%)	Upper Age Target	
		10 years	15 years
Swaziland	90	2	3
Swaziland	70	4	5
Ethiopia	90	7	9
Ethiopia	70	23	25
Nigeria	90	3	3
Nigeria	70	39	38
Equatorial Guinea	90	26	29
Equatorial Guinea	70	8	11
