# Supplementary material for: Demographics, epidemiology and the impact of vaccination campaigns in a measles-free world – Can elimination be maintained?
Source: Vaccine. 2017 Mar 13;35(11):1488–93. doi: 10.1016/j.vaccine.2017.02.008 (PMC5341736; doi:10.1016/j.vaccine.2017.02.008)
Supplement: Supplementary data 1 [file mmc1.pdf]

Supplementary material 1 - Full model description and Sensitivity Analysis

Joaquin M. Prada *et al.*

January 26, 2017

We created an age structured epidemiological MSIRV (Maternally immune - Susceptible - Infected - Recovered - Vaccinated) compartmental model, following methods developed by *Metcalf et al.* [1]. Our simulations require five steps, outlined below: initiating the starting susceptible population size and distribution, forward simulation of the susceptible population through time, evaluation of R_E at each time point, estimation of the integrated epidemic risk, and a simple marginal cost comparison.

1 Initiating the population

We generated simulations based on the urban population of four countries in sub-Saharan Africa; Nigeria, Ethiopia, Equatorial Guinea, and Swaziland. The simulated populations do not strictly represent the countries, but rather populations with similar demographics. We used projections for urban settings only, as the urban populations are more connected between themselves and could be considered a single mixed population. Further, we note that results for rural populations are qualitatively similar (a rural setting is expected to have lower vaccination coverage than an urban setting), and are not discussed further in this manuscript.

The population age structure is based on estimates from "Worldpop" [ref. "http://www.worldpop.org.uk/"]. We stratified the population into 225 age groups (monthly strata up to 15 years of age yearly thereafter). Each strata in the population is further structured as an MSIRV model, with the key element being a matrix that at every time-step (a bi-week, equal to the latent plus infectious period of measles) defines the transition from every possible

epidemiological stage (e.g. maternally immune, susceptible, infected, recovered, or vaccinated) and age combination to every other epidemiological stage and age combination [1]. We assumed seasonal fluctuations in transmission [1], Figure S2 − right; and an R_0 of 15.

To initialize the populations, we need to assign for each age bin, how many people are in each epidemiological status:

1.1 Vaccinated

We assumed that a fraction, P_t, was vaccinated based on the UNICEF/WHO administrative estimates since 1980 and the efficacy of the vaccine (see Table S1 at the end of the document). We also assume that some cohorts received a second opportunity for vaccination through previous SIAs conducted in the populations. All individuals are assumed to have the same probability of being vaccinated. Vaccine efficacy was modelled as a saturated function of age, based on the vaccine efficacy trials by Boulianne *et al.* [2]. Moreover, we limited the maximum efficacy of the vaccine to 97%.

1.2 Recovered

These individuals had measles in the past and thus are no longer susceptible. To model natural exposure to measles, we made the simple assumption of a constant hazard rate of infection with age (measured in months); thus the probability of remaining susceptible declines as an exponential function of age. We assumed a rate of 0.02 for the exponential, which is equivalent to assuming a mean age of infection of 4 years and moved the appropriate proportion of unvaccinated individuals to the recovered status. We also explored sensitivity to variation in the average age of infection, which will change the initial proportion of naturally immunized in the population (see below sensitivity to natural exposure section).

1.3 Infected

Our simulations occur under the assumption that we have achieved elimination, and thus there are no infected individuals at the beginning of the simulation. Infected individuals are seeded at a later stage in the model (see R_E and IER section in the main article).

1.4 Maternally immune

A fraction of the population will have protection due to maternal antibodies. The decay of maternal immunity with age was modeled as a monthly exponential decline with a rate= 0.45 [3], all individuals are assumed to be born with maternal immunity (so maternal protection for the first age bins is 100%).

1.5 Susceptibles

The remainder individuals that are not allocated to any of the previous epidemiological status are assumed to be susceptible.

2 Forward simulations

We simulate the trajectory of the population forward for 15 years, from 2015 to 2030, which involves tracking aging, births and mortality and changes in epidemiological status (loss of maternal immunity and vaccination) in each age group.

Aging can be trivially modeled by assuming the proportion of individuals aging in each time step is one over the size of the age group, measured in bi-weekly time-steps (i.e. 1/2 for the monthly age bins, 1/24 for the yearly age bins). The birth rates were taken from "World Bank" ("http://data.worldbank.org/"), and we assumed the same birth rate of 2015 is maintained throughout the simulation. Mortality in each age group was estimated by interpolating to our age groups the data from the UN Department of Economic and Social Affairs model life tables ("http://esa.un.org/unpd/wpp/Download/Standard/Mortality/"), which is given in 5 year age bins.

Maternal immunity status can only be achieved at birth, and we assume that all newborns have maternal immunity. The loss of maternal immunity, modelled as above, will move individuals to a susceptible state. Vaccination is added from two sources, routine coverage and SIAs, as described in the next paragraph. Susceptible and Maternally Immune individuals can be vaccinated and moved to the vaccinated state, and while recovered individuals could potentially be vaccinated, in our model we consider that both recovered and vaccinated states are fully immunized and thus movements between these two states are irrelevant. Susceptible individuals can also become infected, which will last one time-step (2 weeks) and then moved to the

recovered state. All relevant transitions between epidemiological states are summarized in Figure S1.

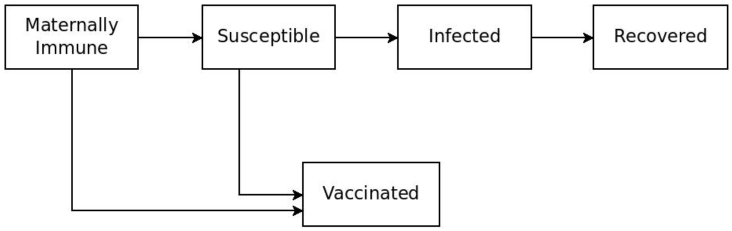

Figure S1: Epidemiological transitions considered in each age bin.

Routine vaccination coverage levels reflected those achieved in 2014 (the most recently available year in the WHO/Unicef database) and remained constant over the simulation time-frame. For SIAs, we explored scenarios reflecting campaigns targeting 3 different age classes (9m to 5y, 9m to 10y, 9m to 15y) occurring every 4 years, which translates into 3 vaccination campaigns in 2019, 2023 and 2027. The frequency of campaigns reflected WHO recommendations from the global measles and rubella strategic plan [4, 5]. Though the intent of campaigns is to vaccinate a large proportion of susceptibles in the target age class, the proportion of susceptibles immunized in these campaigns may differ from the vaccination coverage because of logistical constraints in implementation, under-estimation of the target population size, or heterogeneity in access to vaccination services [6]. Thus, we considered two levels of immunization achieved by these campaigns: 70% and 90%.

In practice, many countries conduct so-called “catch-up” SIAs, which target a wide age range, followed by “follow-up” campaigns, which target a narrower range [7]. We explored scenarios in which the first SIA targeted a wide age range (9m to 10y or 9m to 15y) followed by two “follow-up” campaigns that targeted only children under 5 years. The timing of these 3 SIAs is the same as in the scenarios with the 3 equal ones (first in 2019, then 2023 and 2027). In these scenarios, we assumed that the proportion of immunization achieved in both the “catch-up” and “follow-up” campaigns was consistent, either 70% or 90%. Simulations where the “catch-up” campaign targets individuals from 9m to 5y is redundant with previous settings.

These forward simulations are run under the assumption that elimination has been achieved, which means that there are no infected individuals (additional simulations are run with infected individuals and are explained in the

Integrated Epidemic Risk section). The susceptible population hence will slowly increase based on the number of unvaccinated births (missed by routine coverage) and drastically decrease when the supplementary campaigns are implemented. With the initial susceptible age distributions obtained as described in the previous section, all populations start with a R_E below 1, so the minimal condition for elimination holds.

3 Evaluation of R_E at each time step

Based on the population structure from the forward simulations (projected population size and age distribution of susceptibles), we estimated the effective reproduction number, R_E in each time step for the length of the model run (15 years). R_E is the dominant eigenvalue of the next generation matrix, which can be constructed in each time step and requires the specification of a contact matrix.

Because contact patterns over age are unknown for the countries modelled here, we chose to reflect the contacts between age classes using data from the POLYMOD diary studies based on 8 different European countries [8], Figure S2 – left. The qualitative patterns indicated by POLYMOD are broadly consistent across Europe, and are echoed in a number of other settings [9, 10], and therefore likely to be a reasonable approximation of local patterns.

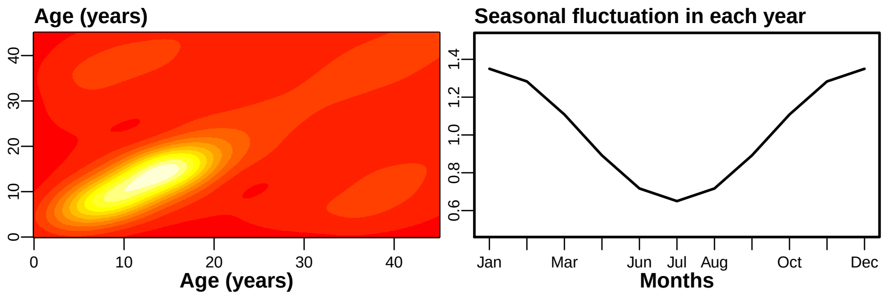

Figure S2: Visual representations of the Who Acquires Infection From Whom (WAIFW) matrix on the left; seasonal forcing as modelled in [1] on the right.

4 Estimation of the Integrated Epidemic Risk (IER)

The effective reproduction number, R_E, is a standard characterization of epidemic potential. However, given that expected outbreak size scales with R_E in a non-linear fashion, it does not fully capture the increased outbreak risk associated with R_E values slightly above 1. We characterize the Integrated Epidemic Risk as the expected outbreak size, divided by the total population, N, and averaged over the simulation time frame, T,

$$IER = \frac{1}{T} \sum_{t=0}^{T} \frac{C_t}{N_t} \tag{1}$$

where C_t is the expected outbreak size after a single introduction at time t and allowing for the potential outbreak to develop over a maximum time of 1 year, N_t is the population at the time of the introduction. We assume that the initial infectious individual is in the highest transmitting age class at that time step (normally an individual younger than 15 years of age), as this represents the worst-case scenario. Estimating the outbreak size in this way assumes that the population is well mixed spatially − while this is unlikely to be the case, the consequence will be over-estimation of outbreak sizes, which will make our results conservative.

Given the non-linear relationship between R_E and epidemic size, the IER reflects better the relatively high risk associated with allowing R_E to increase beyond 1. Thus, a decision-maker should be averse to R_E values slightly over 1, because an outbreak, if it occurred, would be disproportionately large. Averaging over the full time frame balances periods of low risk (i.e. small expected outbreaks) against periods of high risk. To reflect these fluctuating risks, we also calculated the interquartile range across the 15 years, which captures the differences between an introduction at a low and a high risk setting (which depends on the R_E at the time of the introduction). A wide difference in these quantiles indicates either large fluctuations of R_E at low values (i.e. around 1) or relatively small fluctuations at higher R_E values (i.e. R_E between $2-3$).

We can then relate the number of secondary cases arising in each time step to the R_E in the population at the time of the introduction to obtain a utility function, Figure S3. Here, this utility function characterizes how the number of secondary cases varies as the R_E changes − so captures the

Integrated Epidemic Risk. It is of key importance because it quantifies the integrated outbreak risk over coming years and thus enhances the ability of a decision maker to act appropriately, since alternative campaign designs will have variable impacts on changes in R_E [11]. Utility functions have been used in the past in similar settings [12].

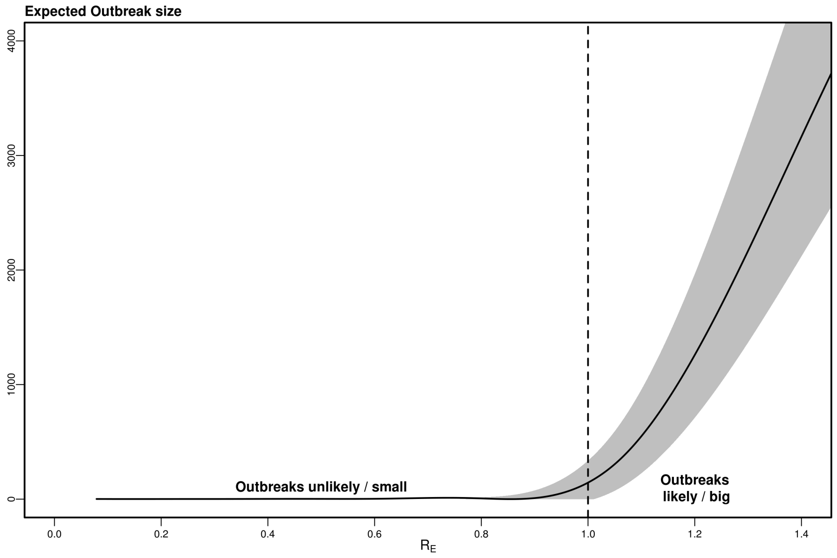

Figure S3: Utility function showing the increase in expected outbreak size as the R_E increases. Vertical dashed line indicates the threshold of $R_E = 1$. Shaded area shows the confidence interval.

5 Simple marginal cost comparison

Higher campaign coverage or wider age targets will result in a greater reduction in susceptibles and lower IER. However, the marginal benefits of increasingly large campaigns should decrease once a campaign is of sufficient coverage to reduce R_E to below 1 and because older individuals are more likely to be previously immunized. Thus we present the impact of larger campaigns in terms of the marginal benefit; i.e. reduction of IER per dose required to implement the campaigns, where the number of doses is used as a simple characterization of campaign cost. SIAs do not generally attempt to discriminate between immune and susceptible individuals within the target

age range. As a result, previously vaccinated individuals (via routine vaccination or in a previous campaign) may be re-vaccinated. The minimum number of doses required for an SIA is thus defined by a combination of the number of individuals in the target age range, and the vaccination coverage the SIA achieves. The true cost of campaigns will depend on operational costs, as well as cost of vaccines and supplies; Vijayaraghavan et al. [5] estimated that vaccine cost and medical supplies account for around 65% of the total cost, while the rest are operational costs. A formal assessment of the operational costs of campaign deployment in each country is beyond the scope of this work, though such an analysis could be incorporated in the framework presented here.

We compared the minimum number of doses needed with the IER (as defined above) for each scenario, showing both the median, as well as the 25th and 75th quantiles, which reflects a lower and higher risk setting respectively. To enable comparisons between the different scenarios, we calculated the relative difference, d_r, in IER using the lowest impact SIA campaign studied (70% immunization of children up to 5 years of age) as the reference (default campaign). As the campaigns increase in coverage or in the extent of the population targeted, the number of vaccine doses required will increase and the number of secondary cases after a single introduction will decrease. By calculating the standardized ratios we can clearly characterize how much a campaign will reduce the number of secondary cases, and at what cost in terms of increased number of doses deployed.

6 Sensitivity to natural exposure

The proportion of recovered individuals, which represents the individuals that had measles and haven't been vaccinated, has been modelled in the past as an exponential increase over time [1] with a rate parameter for the exponential that will affect the average age of infection. We used a rate so as to have a mean age of infection of 4 years as well as exploring a higher rate (mean age of 3.3 years) and a lower rate (mean age of 8.3 years), shown in Figure S4.

The change in the initial proportion of susceptibles is shown in Figure S5. The most significant variation occurs, in all populations except Eq. Guinea, in older age classes (> 25 years of age), while in Eq. Guinea is around $10-15$ years of age. This can be linked to how previous SIAs, vaccine coverage

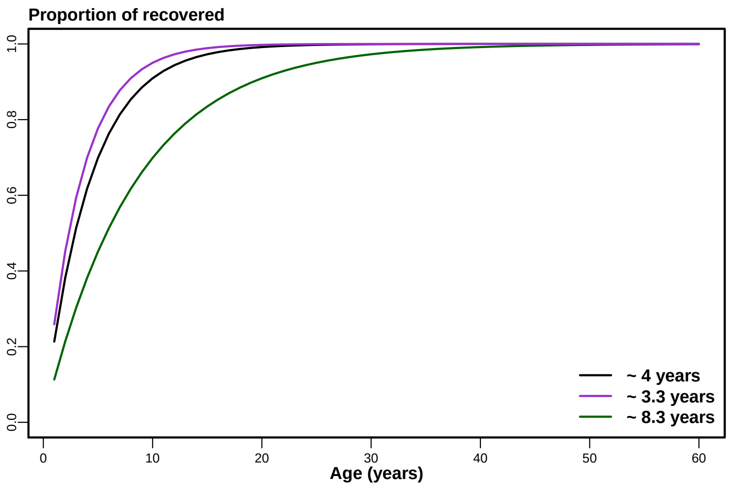

Figure S4: Proportion of the population in each age bin that is recovered, i.e. has had measles in the past and can no longer be infected. The curves were modelled using an exponential function with rate 0.02 (black) for an average age of infection of 4 years; 0.025 (purple) for an average age of infection of 3.3 years; 0.01 (green) for an average age of infection of 8.3 years.

changes and births scale with a bigger susceptible pool. It is important to note that the larger number of susceptible occurs with a higher average age of infection, which is unlikely to happen when coverage is low. Moreover, a low rate parameter in the exponential function, which causes a high mean age of infection in Eq. Guinea, causes the population to be with an R_E over one at the beginning of the simulations, which means that it would not be in an elimination setting and therefore could not be considered further. Due to the low vaccination coverage, it is unlikely that Eq. Guinea has such a high mean age of infection. The results for all the forward simulations considered remain qualitatively similar to the results shown in the main manuscript and are not discussed further.

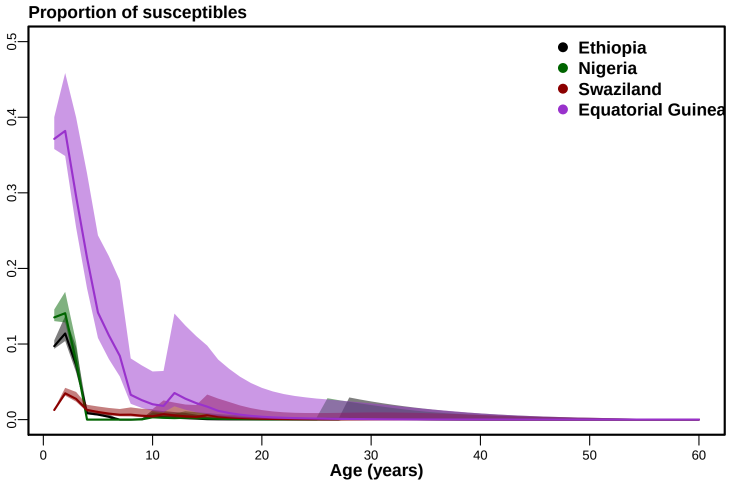

Figure S5: Initial proportion of susceptibles as a function of age. Shaded area indicate the confidence intervals, lines show the value used in the forward simulations (for a rate of the exponential = 0.02).

7 Sensitivity to R_0

In our model, we assumed the value for the R_0 of measles to be 15. We explored here a low and high value for R_0, 12 and 18 respectively, in line with the range reported in [13]. Shown in Figure S6 are the results for Ethiopia, with the rest of the countries studied in the manuscript exhibiting qualitatively similar results.

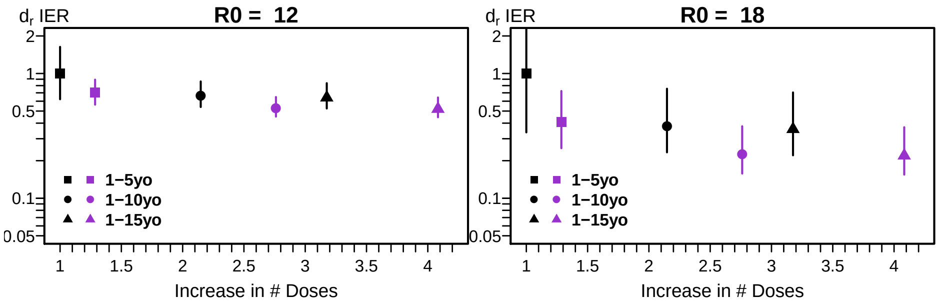

Figure S6: Relative difference in IER (d_r IER) as the SIA campaign increase in coverage and target population in Ethiopia. The reference used is the default SIA campaign (70% coverage, target population up to 5 years).Vertical lines show the interquartile range. The y axis was logged and truncated in the right plot for easier interpretation of the results, y axis limits are different than in Figure 2 of the main manuscript. Black lines indicate 70% coverage SIAs while purple lines show the results for 90% coverage.

As shown in Figure 2 of the main article, qualitatively similar results are obtained across a range of R_0 values. The interquartile range tends to increase with higher R_0, as transmission is higher, and thus the variation in integrated epidemic risk will increase. Extending R_0 further beyond this range does not modify the broad conclusions(not shown), with lower R_0 values giving results in line with highly vaccinated countries (such as Swaziland) and higher R_0 values giving results similar to countries with lower historical routine coverage (such as Eq. Guinea).

8 Alternative contact matrix

Our model was built using the POLYMOD diary studies [8]. However, these studies were originally done in European countries, and thus the contact

structure is likely to be different in Sub-Saharan African countries. We simulated a flat contract matrix (i.e. same contact between all age classes), as an alternative to POLYMOD, shown in Figure S7 are the results for the four countries studied. In general, the integrated epidemic risk is slightly higher using a flat contact matrix, which is expected. However, the qualitative trends are still maintained, IER decreases as coverage / target age range increases, and targeting individuals up to 15 years does not provide significant benefits compared to 10 years, for a given coverage.

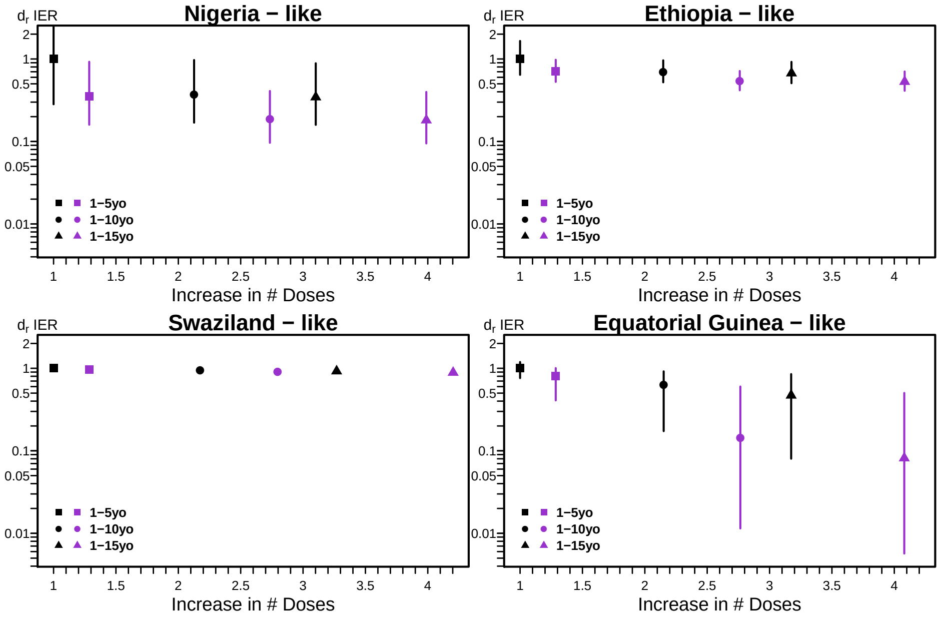

Figure S7: Relative difference in integrated epidemic risk (d_r IER) as the SIA campaign increase in coverage and target population in all four countries modelled. The reference used is the default SIA campaign (70% coverage, target population up to 5 years).Vertical lines show the interquartile range. The y axis was logged. Black lines indicate 70% coverage SIAs while purple lines show the results for 90% coverage.

9 Changing Birth rates

Our simulations in the main paper assume a fixed birth rate for the projections 15 years into the future (from 2015 to 2030), using the most recent

reported birth rates for each country. We explored in this section the effect of allowing birth rate to change over time. To be more precise, we expect birth rates to decline in the four countries studied here [14]. We decided to model that decline using a logistic function, with the lower asymptote being the birth rate needed to maintain a total fertility rate (TFR) of 2.1, which is in line with modernized western countries. The main results of relative difference in IER for the four countries studied are shown in Figure S8.

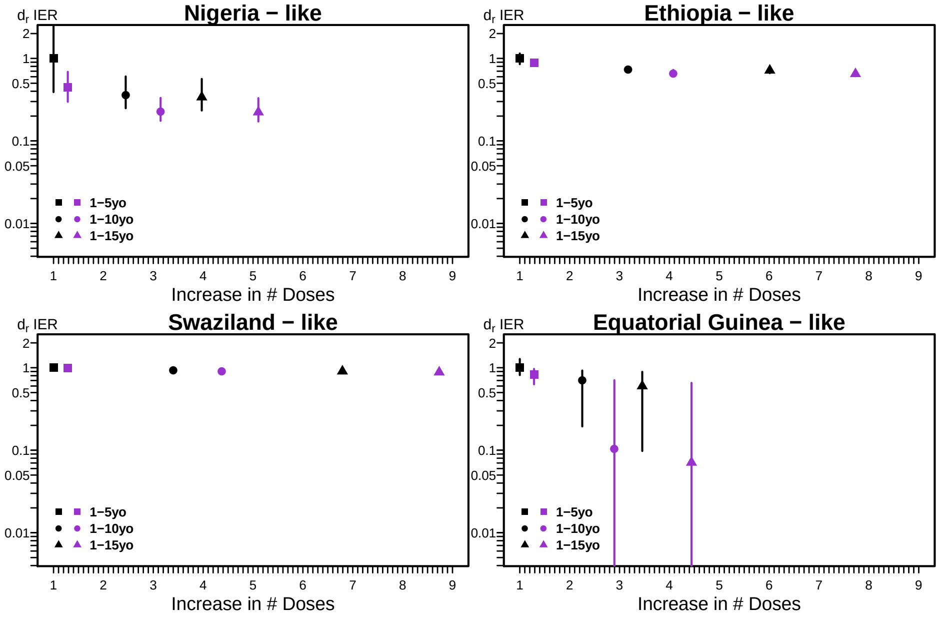

Figure S8: Relative difference in integrated epidemic risk (d_r IER) as the SIA campaign increase in coverage and target population in all four countries modelled. The reference used is the default SIA campaign (70% coverage, target population up to 5 years).Vertical lines show the interquartile range. The y axis was logged. Black lines indicate 70% coverage SIAs while purple lines show the results for 90% coverage.

The main difference in our results is the change in relative number of doses. Because the birth rates are lower, the total number of doses used is lower (e.g. The basic 70% coverage, up to 5 years SIAs campaign in an Ethiopia-like population requires slightly over 1M doses, while in the main manuscript it was well over 3M). However, the relative change in doses as we increase coverage / target upper age increases (note the change in the

x-axis in Figure S8 compared to Figure 2 of the main manuscript). In some countries, such as the Eq. Guinea-like population, the interquartile range also increases, this is also expected, as in a smaller population, due to the reduced birth rate, the relative effect of a few infected individuals is more significant. Nevertheless, our general conclusions on the relative change in integrated epidemic risk as SIA campaigns increase in coverage / target upper age are qualitatively the same, where increase target age for SIAs up to 15 years, in a setting such as ours with recurrent campaigns every 4 years, does not provide significant benefits over targeting individuals up to 10 years.

10 Historical Vaccination Data

The tables below summarize the vaccination data used to initialize the populations, both routine and previous SIAs [15]. For missing data on routine vaccination coverage, we used smoothing splines with six degrees of freedom to interpolate the values. For the historical SIAs, some of the data is from administrative coverage, we assumed an overlap equal to 1, which means that only the highest coverage in each age bin is taken into account, the maximum coverage was also limited to 100%. Uncertainty in the historical vaccination data, particularly the SIAs campaigns is accounted for in the sensitivity analysis to natural exposure, which effectively moves individuals out of the susceptible class.

Table S1: Historical vaccination coverage for the 4 countries considered, during the more recent 20 years. Older values are available online at the WHO website. The value for the most recent year, 2014, is used in the forward simulations as routine vaccine coverage achieved.

Coverage (%) / Year	2014	2013	2012	2011	2010	2009	2008	2007	2006	2005
Ethiopia	81	78	80	82	81	75	74	65	63	59
Nigeria	73	73	78	93	85	81	68	86	99	34
Eq. Guinea	43	41	34	50	51	77	76	37	25	24
Swaziland	97	97	88	98	94	72	69	58	57	60

Coverage (%) / Year	2004	2003	2002	2001	2000	1999	1998	1997	1996	1995
Ethiopia	56	44	42	40	37	32	46	33	34	38
Nigeria	40	-	-	-	30	-	26	38	38	44
Eq. Guinea	33	36	51	52	19	24	-	82	61	61
Swaziland	70	94	72	72	80	82	82	92	82	94

Table S2: Historical supplementary immunization activities (SIAs) in the 4 countries considered, including coverage and age range. The data were originally sourced from WHO [15].

Country	Year	Coverage (%)	Age range (months)
Ethiopia	2000	95	9 − 59
Ethiopia	2001	76	9 − 59
Ethiopia	2002	98	9 − 168
Ethiopia	2003	91	6 − 168
Ethiopia	2004	84	6 − 168
Ethiopia	2005	92	9 − 59
Ethiopia	2005	69	6 − 168
Ethiopia	2006	87	9 − 59
Ethiopia	2007	96	6 − 59
Ethiopia	2008	92	6 − 59
Ethiopia	2009	95	6 − 59
Ethiopia	2010	91	6 − 59
Ethiopia	2010	100	9 − 47
Ethiopia	2011	98	9 − 47
Ethiopia	2011	96	6 − 168
Ethiopia	2013	98	9 − 59
Nigeria	2005	95	9 − 180
Nigeria	2006	83	9 − 180
Nigeria	2007	89	9 − 59
Nigeria	2007	78	60 − 132
Nigeria	2008	97	9 − 59
Nigeria	2011	100	9 − 59
Nigeria	2013	26	6 − 59
Nigeria	2013	100	9 − 59
Eq. Guinea	2003	17	0 − 23
Eq. Guinea	2005	44	9 − 180
Eq. Guinea	2009	80	12 − 59
Eq. Guinea	2011	50	9 − 47
Eq. Guinea	2012	58	9 − 59
Swaziland	2002	81	9 − 59
Swaziland	2006	91	9 − 59
Swaziland	2009	96	9 − 47
Swaziland	2010	90	9 − 59
Swaziland	2013	97	6 − 59

References

[1] C. J. E. Metcalf, J. Lessler, P. Klepac, A. Morice, B. Grenfell, and O. N. Bjornstad, “Structured models of infectious disease: inference with discrete data,” *Theoretical Population Biology*, vol. 82, no. 4, pp. 275–82, 2012.

[2] N. Boulianne, G. De Serres, S. Ratnam, B. Ward, J. Joly, and B. Duval, “Measles, mumps, and rubella antibodies in children 5-6 years after immunization: effect of vaccine type and age at vaccination,” *Vaccine*, vol. 13, pp. 1611–1616, 1995.

[3] J. Lessler, W. J. Moss, S. A. Lowther, and D. a. T. Cummings, “Maintaining high rates of measles immunization in Africa,” *Epidemiology & Infection*, vol. 139, pp. 1039–1049, July 2011.

[4] W. H. Organization, *Global measles and rubella strategic plan : 2012-2020*.

[5] M. Vijayaraghavan, F. Lievano, L. Cairns, L. Wolfson, R. Nandy, A. Ansari, A. Golaz, T. Mashal, and P. Salama, “Economic Evaluation of Measles Catch-Up and Follow-Up Campaigns in Afghanistan in 2002 and 2003,” *Disasters*, vol. 30, pp. 256–269, June 2006.

[6] J. Lessler, C. J. E. Metcalf, R. F. Grais, F. J. Luquero, D. A. T. Cummings, and B. T. Grenfell, “Measuring the Performance of Vaccination Programs Using Cross-Sectional Surveys: A Likelihood Framework and Retrospective Analysis,” *PLoS Med*, vol. 8, p. e1001110, Oct. 2011.

[7] R. Biellik, S. Madema, A. Taole, A. Kutsulukuta, E. Allies, R. Eggers, N. Ngcobo, M. Nxumalo, A. Shearley, E. Mabuzane, E. Kufa, and J.-M. Okwo-Bele, “First 5 years of measles elimination in southern Africa: 19962000,” *The Lancet*, vol. 359, pp. 1564–1568, May 2002.

[8] J. Mossong, N. Hens, M. Jit, P. Beutels, K. Aranen, R. Mikolajczyk, M. Massari, S. Salmaso, G. Scalia Tomba, J. Wallinga, J. Heijne, M. Sadkowska-Todys, M. Rosinska, and W. J. Edmunds, “Social Contacts and Mixing Patterns Relevant to the Spread of Infectious Diseases,” *PloS Medicine*, vol. 5, p. e74, 2008.

[9] J. M. Read, J. Lessler, S. Riley, S. Wang, L. J. Tan, K. O. Kwok, Y. Guan, C. Q. Jiang, and D. A. T. Cummings, "Social mixing patterns in rural and urban areas of southern China," *Proceedings of the Royal Society of London B: Biological Sciences*, vol. 281, p. 20140268, June 2014.

[10] P. Rohani, X. Zhong, and A. A. King, "Contact Network Structure Explains the Changing Epidemiology of Pertussis," *Science*, vol. 330, pp. 982–985, Nov. 2010.

[11] W. Edwards, R. F. M. Jr, and D. v. Winterfeldt, *Advances in Decision Analysis: From Foundations to Applications*. Cambridge University Press, July 2007.

[12] K. Shea, M. J. Tildesley, M. C. Runge, C. J. Fonnesbeck, and M. J. Ferrari, "Adaptive Management and the Value of Information: Learning Via Intervention in Epidemiology," *PLoS Biol*, vol. 12, p. e1001970, Oct. 2014.

[13] D. N. Durrheim, N. S. Crowcroft, and P. M. Strebel, "Measles the epidemiology of elimination," *Vaccine*, vol. 32, no. 51, pp. 6880 – 6883, 2014.

[14] J. Bongaarts and J. Casterline, "Fertility Transition: Is sub-Saharan Africa Different?," *Population and development review*, vol. 38, pp. 153–168, Feb. 2013.

[15] R. T. Perry, J. S. Murray, M. Gacic-Dobo, A. Dabbagh, M. N. Mulders, P. M. Strebel, J.-M. Okwo-Bele, P. A. Rota, and J. L. Goodson, "Progress toward regional measles elimination - worldwide, 2000-2014," *MMWR*, vol. 64, pp. 1246–1251, 2015.
