# Supplementary material for: Demographics, epidemiology and the impact of vaccination campaigns in a measles-free world – Can elimination be maintained?
Source: Vaccine. 2017 Mar 13;35(11):1488–93. doi: 10.1016/j.vaccine.2017.02.008 (PMC5341736; doi:10.1016/j.vaccine.2017.02.008)
Supplement: Supplementary data 2 [file mmc2.doc]

Table SM2: Estimated number of individuals that are vaccinated twice (2V), three times (3V) and four times (4V) after the 3 SIA campaigns and the percentage of the total population they represent in brackets. *k* is used to symbolize 1000 individuals; M to symbolize a million. Numbers have been rounded for readability. Campaigns with target upper age of 5 years do not generate individuals vaccinated more than twice because the SIAs are spaced 4 years and the target population is from 1 to 5 years of age.

|  |  | Upper Age Target | | |
| --- | --- | --- | --- | --- |
| Country | Coverage | 5 years | 10 years | 15 years |
| Swaziland | 90% | 2V=36k (9.1%)  3V=0 (0%)  4V=0 (0%) | 2V=39k (10%)  3V=32k (8.2%)  4V=7.1k (1.8%) | 2V=39k (10%)  3V=36k (9%)  4V=38k (9.7%) |
| Swaziland | 70% | 2V=27k (7%)  3V=0 (0%)  4V=0 (0%) | 2V=36k (9.3%)  3V=20k (5.2%)  4V=3.3k (0.85%) | 2V=39k (9.9%)  3V=26k (6.7%)  4V=18k (4.5%) |
| Ethiopia | 90% | 2V=1.2M (7.6%)  3V=0 (0%)  4V=0 (0%) | 2V=1.33M (8.5%)  3V=1.26M (8%)  4V=287k (1.8%) | 2V=1.34M (8.6%)  3V=1.4M (8.9%)  4V=1.48M (9.5%) |
| Ethiopia | 70% | 2V=920k (6%)  3V=0 (0%)  4V=0 (0%) | 2V=1.25M (8%)  3V=775k (5%)  4V=130k (0.8%) | 2V=1.35M (8.7%)  3V=1M (6.5%)  4V=690k (4.5%) |
| Nigeria | 90% | 2V=7.2M (8.7%)  3V=0 (0%)  4V=0 (0%) | 2V=8.35M (9.8%)  3V=8.3M (9.7%)  4V=1.85M (2.2%) | 2V=8.45M (9.9%)  3V=9.2M (10%)  4V=9.7M (11%) |
| Nigeria | 70% | 2V=5.76M (6.7%)  3V=0 (0%)  4V=0 (0%) | 2V=7.9M (9.2%)  3V=5M (5.9%)  4V=850k (1%) | 2V=8.6M (10%)  3V=6.6M (7.9%)  4V=4.5M (5.2%) |
| Equatorial Guinea | 90% | 2V=3.8k (4.9%)  3V=0 (0%)  4V=0 (0%) | 2V=4.6k (5.9%)  3V=6.9k (8.8%)  4V=1.6k (2.1%) | 2V=4.7k (6%)  3V=7.6k (9.7%)  4V=8.1k (10%) |
| Equatorial Guinea | 70% | 2V=3k (3.8%)  3V=0 (0%)  4V=0 (0%) | 2V=4.6k (5.9%)  3V=3.9k (5%)  4V=720 (0.9%) | 2V=5.2k (6.6%)  3V=5.2k (6.6%)  4V=3.6k (4.6%) |
